# Polyphenols in Ilex latifolia Thunb. inhibit human lung cancer cell line A549 by regulation of the PI3K-Akt signaling pathway

**DOI:** 10.1186/s12906-022-03568-3

**Published:** 2022-03-23

**Authors:** Jing Chen, Yesheng Du, Yanyan Long, Dan Tao, Mengyu Hu, Yong Jiang, Yue Wan, Dingyi Yang

**Affiliations:** 1 Chongqing Key Laboratory of Translational Research for Cancer Metastasis and Individualized Treatment, Chongqing University Cancer Hospital, Chongqing, 400030 China; 2 Key Laboratory for Biorheological Science and Technology of Ministry of Education (Chongqing University), Chongqing University Cancer Hospital, Chongqing, 400030 China

**Keywords:** Lung cancer, MTT, PI3K-Akt signaling pathway, Polyphenols, *Ilex latifolia* Thunb

## Abstract

**Background:**

The leaves of the plant *Ilex latifolia* Thunb. can be made into Kuding tea, which is a drink rich in polyphenols. This study aimed to observe the effect of *Ilex latifolia* Thunb. polyphenols (ILTPs) on human lung cancer cell line A549 (A549 cells) by regulating the phosphatidylinositol 3-kinase/protein kinase B (PI3K/Akt) signaling pathway.

**Methods:**

*In vitro* cultured cells were treated with ILTPs; the proliferation of A549 cells and BEAS-2B human normal lung epithelial cells (Beas-2B cells) was observed using the 3-(4,5-dimethylazol-2-yl)-2,5-diphenyltetrazolium bromide (MTT) assay, and the survival status of A549 cells was observed by fluorescence staining. The expression of A549 cells was observed by quantitative polymerase chain reaction (qPCR) assay and Western blot analysis, while the compound composition of ILTPs was detected using high-performance liquid chromatography (HPLC).

**Results:**

The experimental results showed that the proliferation of Beas-2B cells was unaffected by treatment with 0–500 μg/mL of ILTPs, whereas the decreased proliferation of A549 cells was observed with the increasing concentrations of ILTPs. Additionally, ILTPs elevated the levels of lactate dehydrogenase (LDH) and reactive oxygen species (ROS) and promoted apoptosis in A549 cells. The results of qPCR experiments showed that ILTPs upregulated caspase-9 mRNA expression and downregulated phosphatidylinositol 3-kinase (PI3K), protein kinase B (Akt), mammalian target of rapamycin (mTOR), B-cell lymphoma-2 (Bcl-2), nuclear factor-κB (NF-κB), vascular endothelial growth factor (VEGF), hypoxia-inducible factor-1 alpha (HIF-1α), and cyclooxygenase-2 (COX-2) expression in A549 cells. The Western blot analysis results also showed that ILTPs could reduce the protein expression of PI3K and Akt. The HPLC results showed that the main compounds present in the ILTPs were rutin, kaempferol, isochlorogenic acid A, isochlorogenic acid B, and isochlorogenic acid C.

**Conclusions:**

Thus, this study indicated that the polyphenols of *I. latifolia* act as a class of natural functional food materials that potently suppress cancer by exerting their inhibitory effects on A549 cell proliferation through five key polyphenolic compounds.

**Supplementary Information:**

The online version contains supplementary material available at 10.1186/s12906-022-03568-3.

## Background

The leaves of *Ilex latifolia* Thunb. are harvested from plants mainly distributed in southwest and south China. The leaves are dried and brewed into a tea that is consumed as a traditional natural health drink commonly known as Kuding tea in China. The Kuding tea contains more than 200 components, such as saponins, amino acids, vitamin C, polyphenols, flavonoids, caffeine, and protein [1]. *I. latifolia* has the functions of clearing and relieving heat, moistening the throat and relieving cough, reducing blood pressure and weight, inhibiting and preventing cancer, activating blood vessels, and exerting anti-aging effects [2]. Analysis revealed that *I. kudingcha* contains nearly 6% polyphenols [3]. The polyphenols in the tea can remove harmful free radicals, block lipid peroxidation, increase the activity of enzymes in the human body, exert anti-mutation and anticancer effects, and thus can be used for the prevention and auxiliary treatment of various cancers such as gastric and colon cancers [4].

Lung cancer is a common primary cancer. The incidence rate and mortality rate of lung cancer rank second and first, respectively, among those of all other cancer types [5]. The incidence rate of lung cancer has increased significantly over the past 50 years. The incidence and mortality rates of lung cancer are the highest among all cancer types, with these rates being the second highest among females [6]. Studies have shown that the polyphenols from green tea, apples, seaweed, and rice husks can help in inhibiting lung cancer *in vitro* or *in vivo* [7, 8].

The expression of the phosphatidylinositol 3-kinase/protein kinase B (PI3K/Akt) signaling pathway is abnormally activated in the occurrence and development of some cancers. The two most widely discovered mechanisms of PI3K/Akt activation in human cancer are triggered by receptor tyrosine kinase and somatic mutations in specific elements of signaling pathways [9]. The stimulation and promotion of the PI3K pathway may have a negative impact on cancer treatment, and, therefore, the inhibition of PI3K may inhibit cancer development [10]. Abnormal stimulation of the PI3K/Akt pathway is related to tumor growth, angiogenesis, and survival [11], in which the loss of function of tumor suppressor gene phosphatase and tensin homolog deleted on chromosome ten (PTEN) is commonly seen in human tumors and leads to the stimulation of the PI3K/Akt pathway [12]. The key expression of the PI3K/Akt pathway, which includes PI3K, Akt, and mammalian target of rapamycin (mTOR), has been confirmed to have a clinical targeted therapeutic effect [13].

This study verified the inhibitory effect of *I. latifolia* polyphenols on A549 cells by regulating the PI3K/Akt signaling pathway *in vitro*. Moreover, *I. latifolia* polyphenols were analyzed to elucidate the relationship between the active components of *I. latifolia* polyphenols and the regulation of the PI3K/Akt signaling pathway in lung cancer.

## Methods

### Preparation of *I. latifolia* Thunb. polyphenol extract

After freeze-drying, the *I. latifolia* leaves (Yuqing Lvye tea processing factory, Zunyi, Guizhou, China) were crushed, 70% ethanol (v/v) was added according to the ratio of liquid to material 20:1, and the mixture was heated in a water bath at 60°C for 3 h. The ethanol extract was passed through the FL-3 macroporous resin (Shanghai Yiji Biology Co., Ltd., Shanghai, China), the filtrate was collected, and the extract was obtained by rotary distillation of the filtrate. After freeze-drying the extract, the dried product obtained was a fine powder.

### Analysis of *I. latifolia* Thunb. polyphenols extract composition

High-performance liquid chromatography (HPLC, UltiMate3000 HPLC System, Thermo Fisher Scientific, Inc., MA, USA) was used to determine the composition of the flavonoids in *I. latifolia* leaves. The chromatographic conditions were as follows: C18 column (4.6 mm × 150 mm, 2.6 μm); mobile phase A—0.5% acetic acid solution, B—acetonitrile; flow rate, 0.6 mL/min; column temperature, 30°C; detector, ultraviolet visible; detection wavelength, 359 nm; and injection volume, 10 μL. The flavonoid quantitation in mg/g was calculated by the external standard method: Mx = Cr × Ax/Ar × C, where Cr (mg/mL) denotes the mass concentration of the standard, Ax the peak area of the sample, Ar the peak area of the standard, and C (1.0 mg/mL) the concentration of the sample stock solution.

### Culture of cancer cells

The A549 cells and BEAS-2B human normal lung epithelial cells (Beas-2B cells) were used in this study. The Roswell Park Memorial Institute (RPMI)-1640 medium (Invitrogen, CA, USA) was added with 10% fetal bovine serum (v/v) and 1% penicillin–streptomycin (v/v). The prepared medium was used to culture A549 cells and Beas-2B cells (Shanghai Yaji Biotechnology Co., Ltd., Shanghai, China) at 37°C and under a 5% CO_2_ atmosphere, and the medium was changed every 2 days. The cells in the control group were not treated with *Ilex latifolia* Thunb. polyphenols (ILTPs), while the treated cells received 0–500 μg/mL ILTPs.

### Detection of cell proliferation

The cell proliferation was detected using the 3-(4,5-dimethylazol-2-yl)-2,5-diphenyltetrazolium bromide (MTT) assay. For this, 1 × 10^4^/mL cells were seeded into 96-well plates, 180 μL per well, and cultured for 24 h at 5% CO_2_ and 37°C. After the cells adhered to the plate walls, 20 μL of LLTP solution at different concentrations was added to each well. After incubation for 48 h, the medium was discarded, 200 μL of MTT reagent (5 mg/mL, Solarbio, Beijing, China) was added to each well, and the cells were cultured for 4 h. After the end of incubation, the supernatant was discarded, 200 μL of dimethyl sulfoxide was added to each well, and the plate was oscillated for 30 min. The optical density (OD) value of each well was measured at 490 nm, and the inhibition rate of cell proliferation was calculated. The proliferation inhibition rate (%) = (OD_control well_ - OD_sample well_)/OD_control well_ × 100 [14].

### Determination of lactate dehydrogenase levels in the cell culture medium

After the cells were treated according to the method described in the previous section, the medium was collected and the lactate dehydrogenase (LDH) level in the medium was determined using a kit according to the manufacturer’s instructions (Nanjing Jiancheng Bioengineering Institute, Nanjing, Jiangsu, China).

### Detection of apoptosis of cancer cells by flow cytometry

The A549 cells in the logarithmic growth phase were treated with 125, 250, and 500 μg/mL LLTPs for 48 h. Then, the cells were washed with phosphate-buffered saline (PBS) three times, suspended in annexin V–FITC binding solution, mixed with annexin V–FITC and propidium iodide staining solution (Thermo Fisher Scientific, Inc.), and cultured at 4°C in the dark for 15 min. Finally, the apoptosis of cancer cells was analyzed by flow cytometry (FACSCalibur, BD, NJ, USA) [15].

### Detection of reactive oxygen species levels in cancer cells by flow cytometry

The A549 cells treated with nuciferine were collected, washed three times with PBS, resuspended in RPMI-1640 medium containing 10 μmol/L 2',7'-dichlorodihydrofluorescein diacetate at 37°C for 20 min, washed three times with PBS after incubation, resuspended in RPMI-1640 medium, and the fluorescence intensity of each test sample was assessed using a flow cytometer (FACSCalibur, BD, NJ, USA), with the mean fluorescence intensity (MFI) representing the content of reactive oxygen species (ROS) [16].

### Quantitative polymerase chain reaction assay

The total RNA was extracted using a kit, and the concentration and purity of RNA were determined using a microspectrophotometer (Nano 300, All for Life Science, Hangzhou, Zhejiang, China). Reverse transcription of RNA into cDNA was performed using a Reverse Aid First Strand cDNA synthesis kit (Tiangen Biotech Co., Ltd., Beijing, China). Hieff quantitative polymerase chain reaction (qPCR) SYBR Green Master Mix (High Rox Plus), and StepOne Plus real-time PCR system (Thermo Fisher Scientific, Inc.) were used to measure the expression levels of target genes. The qPCR primers used are listed in Table 1. qPCR was carried out under the following cycle conditions: pre-denaturation at 95°C for 3 min, and then denaturation for 15 s at 95°C for 60 cycles; annealing at 55°C for 30 s; denaturation at 95°C for 30 s; and annealing at 55°C for 35 s. β-Actin was used for target gene expression, and the relative intensity of expression was calculated by the 2^−ΔΔCt^ method [17].

**Table 1:** Sequences of primers used in this study.

Gene Name	Sequence
*PI3K*	Forward: 5’-CTGCCTGCGACAGATGAGTG-3’ Reverse: 5’-TCCGATTACCAAGTGCTCTTTC-3’
*Akt*	Forward: 5’-AGCGACGTGGCTATTGTGAAG-3’ Reverse: 5’-GCCATCATTCTTGAGGAGGAAGT-3’
*mTOR*	Forward: 5’-ACAACTTTGGTATCGTGGAAGG-3’ Reverse: 5’-GCCATCACGCCACAGTTTC-3’
*Bcl-2*	Forward: 5’-ATGTGTGTGGAGAGCGTCAACC-3’ Reverse: 5’-CAGAGACAGCCAGGAGAAATCAA-3’
*Caspase-9*	Forward: 5’-CTCAGACCAGAGATTCGCAAAC-3’ Reverse: 5’-GCATTTCCCCTCAAACTCTCAA-3’
*NF-κB*	Forward: 5’-GAAGCACGAATGACAGAGGC-3’ Reverse: 5’-GCTTGGCGGATTAGCTCTTTT-3’
*VEGF*	Forward: 5’-TGCCCACTGAGGAGTCCAAC-3’ Reverse: 5’-TGGTTCCCGAAACGCTGAG-3’
*HIF-1α*	Forward: 5’-ATTCCAGCAGACTCAAATACAAGA-3’ Reverse: 5’-GACTCAAAGCGACAGATAACACG-3’
*COX-2*	Forward: 5’-CTGGCGCTCAGCCATACAG-3’ Reverse: 5’-CGCACTTATACTGGTCAAATCCC-3’
*β-actin*	Forward: 5’-TCAAGAAGGTGGTGAAGCAGG-3’ Reverse: 5’-AGCGTCAAAGGTGGAGGAGTG-3’

### Western blot analysis

The cells were treated in the same way as the qPCR assay. Then, 1 mL of pre-cooled lysate was added to the treatment cells, incubated on ice for 15 min, and then centrifuged at 4°C and 10,000 rpm for 15 min. After centrifugation, the supernatant was removed, and the bicinchoninic acid method was used to determine the protein content. The protein samples were separated using 10% sodium dodecyl sulfate–polyacrylamide gel electrophoresis (Thermo Fisher Scientific), and the protein in the polyacrylamide gel was transferred to the nitrocellulose membrane. The nitrocellulose membrane was blocked with a 5% skimmed milk powder solution at room temperature for 1 h and incubated with the primary antibody (Thermo Fisher Scientific) overnight at 4°C on a shaker. Subsequently, the membrane was washed three times with PBS and Tween 20 (PBST) for 5 min each. Then, the secondary antibody (Thermo Fisher Scientific) was added, the membrane was incubated on a shaker for 2 h, and washed with PBST three times for 5 min each time to enhance the luminescence, development, and imaging effects of the chemiluminescent agent (iBright, Thermo Fisher Scientific) [17].

### Statistical analysis

SPSS v17.0 and GraphPad Prism 7 software were used to analyze the data. The experimental results were expressed as mean ± standard deviation. One-way analysis of variance and *t* test were used to analyze the statistical difference at the level of *P* <0.05. All experiments were repeated three times.

## Results

### Chemical composition of ILTPs

**Fig. 1:**
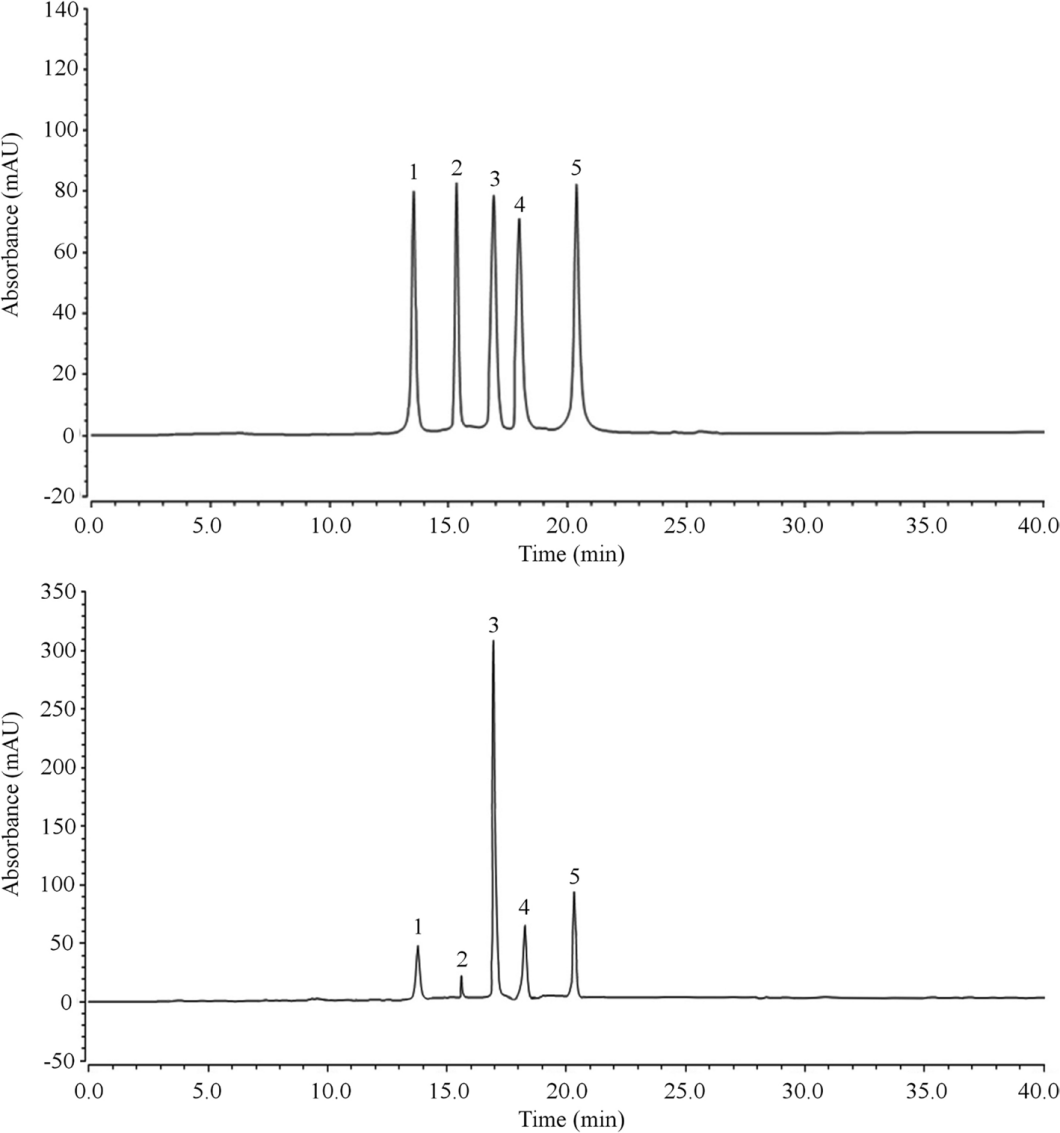
Analysis of ILTPs component. (**A**) Standard chromatograms; (**B**) ILTPs chromatograms. 1: rutin, 2: kaempferol, 3: isochlorogenic acid B, 4: isochlorogenic acid A, 5: isochlorogenic acid C

The analysis indicated that ILTPs were composed of five polyphenols, (i) rutin, (ii) kaempferol, (iii) isochlorogenic acid A, (iv) isochlorogenic acid B, and (v) isochlorogenic acid C (Fig. 1) at 216.35, 167.28, 102.52, 88.12, and 128.36 mg/g (mg/g of ILTPs extract), respectively.

### Cytotoxic effect of ILTPs

**Fig. 2:**
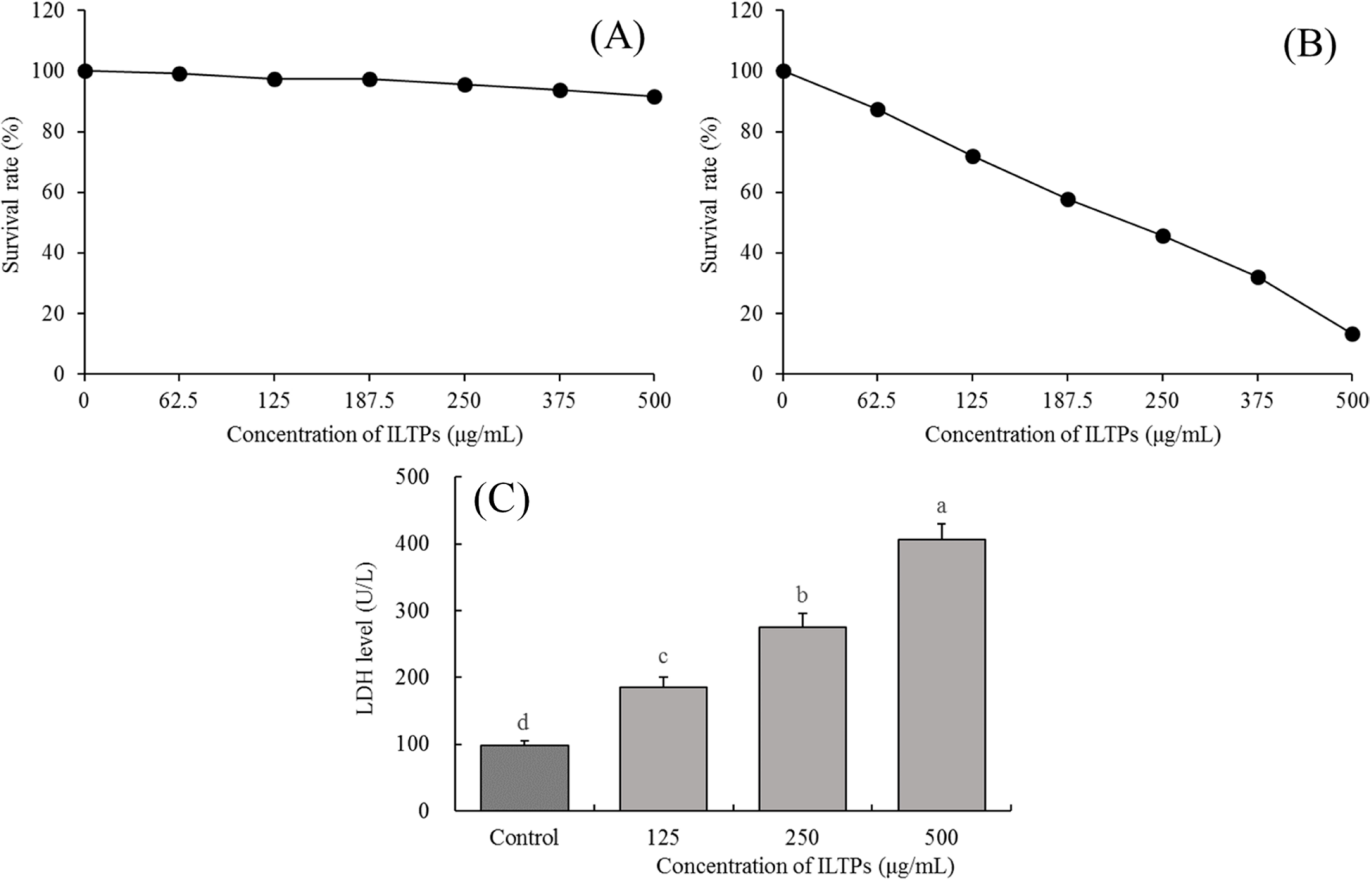
Survival rate of ILTPs treated (**A**) BEAS-2B human normal lung epithelial cells, (**B**) A549 lung cancer cells and (**C**) LDH level of A549 lung cancer cells culture medium (*n*=6). Duncan multiple range test showed that a-d of different letters showed significant difference in the mean value of each group (*P* < 0.05), the same letter indicates that there is no significant difference in the average value of each group (*P* > 0.05)

Figure 2A and B shows that at the concentration of 0–500 μg/mL, ILTPs had only a slight effect on the growth and proliferation of Beas-2B cells. However, at the same concentration, ILTPs inhibited the growth and proliferation of A549 cells, and the inhibition rate positively correlated with the concentration of ILTPs. The results showed that ILTPs had little effect on normal lung cells with no toxicity, but an inhibitory effect was observed on the proliferation of lung cancer cells. At concentrations of 125, 250, and 500 μg/mL, ILTPs inhibited the proliferation of A549 cells by 28.10%, 54.16%, and 86.74%, respectively (Table 2). Based on the aforementioned experimental results, 125, 250, and 500 μg/mL concentrations of ILTPs were used for further experiments. As shown in Fig. 2C, the LDH level in the cell culture medium of the control group was the lowest (97.82 U/L). With the increase in the concentration of ILTPs, the LDH level in the cell culture medium also increased. The LDH level in the culture medium of A549 cells treated with 125, 250, and 500 μg/mL ILTPs was 186.05, 274.88, and 406.73 U/L, respectively.

**Table 2:** Inhibitory effect of different concentrations of ILTPs on proliferation of A549 lung cancer cells (*n*=6)

Group	OD_490_ (Concentration of ILTPs, μg/mL)			Cell growth inhibition rate (%)		
	125	250	500	125	250	500
Control	0.445±0.005^a^			/		
ILTPs	0.320±0.011^b^	0.204±0.007^c^	0.059±0.005^d^	28.10±2.51^C^	54.16±2.02^B^	86.74±1.76^A^

The experimental results are mean ± standard deviation. Duncan multiple range test showed that A-D and a-c of different letters showed significant difference in the mean value of each group (*P* < 0.05), the same letter indicates that there is no significant difference in the average value of each group (*P* > 0.05)

### Apoptosis of A549 cells

**Fig. 3:**
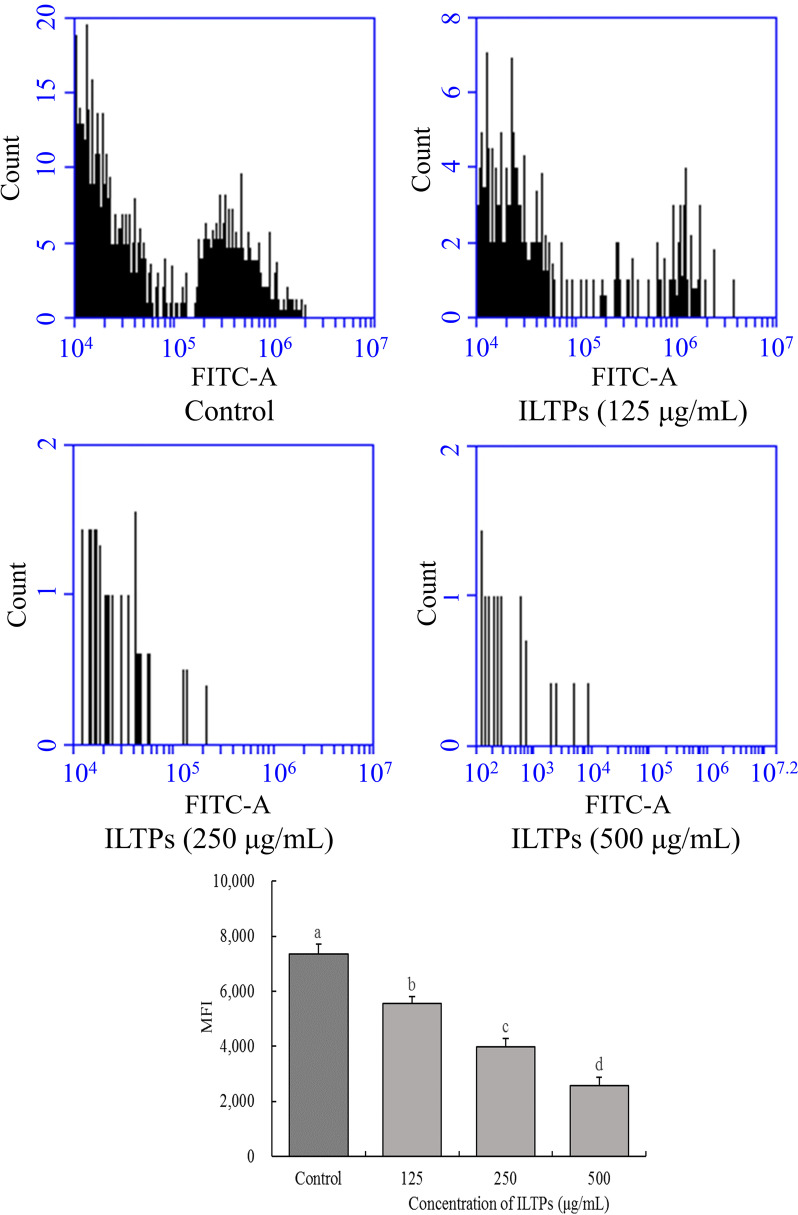
Effects of ILTPs on apoptosis of A549 lung cancer cells (*n*=3). Duncan multiple range test showed that a-d of different letters showed significant difference in the mean value of each group (*P* < 0.05), the same letter indicates that there is no significant difference in the average value of each group (*P* > 0.05)

As shown in Fig. 3, 500 μg/mL of ILTPs (42.92%) significantly induced the apoptosis of A549 cells compared with the control group. Similarly, A549 cells treated with 125 (16.35%) and 250 μg/mL (29.41%) ILTPs also showed apoptosis, and the number of apoptotic cells was higher than that in the control group, but less than that observed in the group treated with 500 μg/mL ILTPs. Also, the concentration of ILTP treatment positively correlated with the effect of apoptosis induction. The apoptosis-inducing effect of ILTPs on A549 cells positively correlated with the concentration of ILTPs.

### ROS levels in A549 cells

**Fig. 4:**
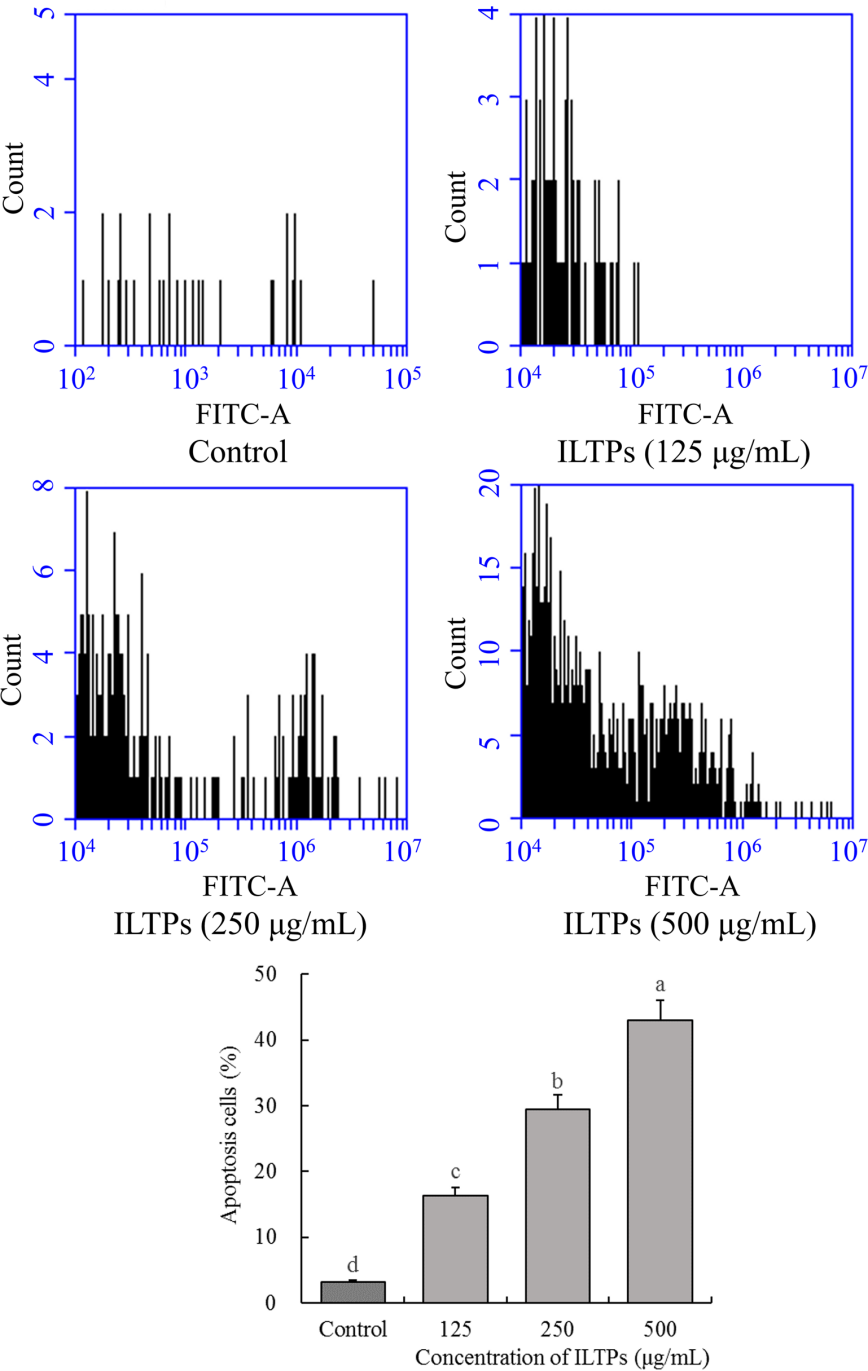
Effects of ILTPs on ROS level in A549 lung cancer cells (*n*=3). Duncan multiple range test showed that a-d of different letters showed significant difference in the mean value of each group (*P* < 0.05), the same letter indicates that there is no significant difference in the average value of each group (*P* > 0.05)

The flow cytometry results shown in Fig. 4 revealed that the MFI of the 125, 250, and 500 μg/mL ILTP groups was significantly lower than that of the control group (*P* < 0.05). The higher the ILTP concentration, the lower the fluorescence intensity, indicating a lower ROS level.

### Expression of PI3K, Akt, mTOR, Bcl-2, caspase-9, NF-κB, VEGF, HIF-1α, and COX-2 mRNA in A549 cells

**Fig. 5:**
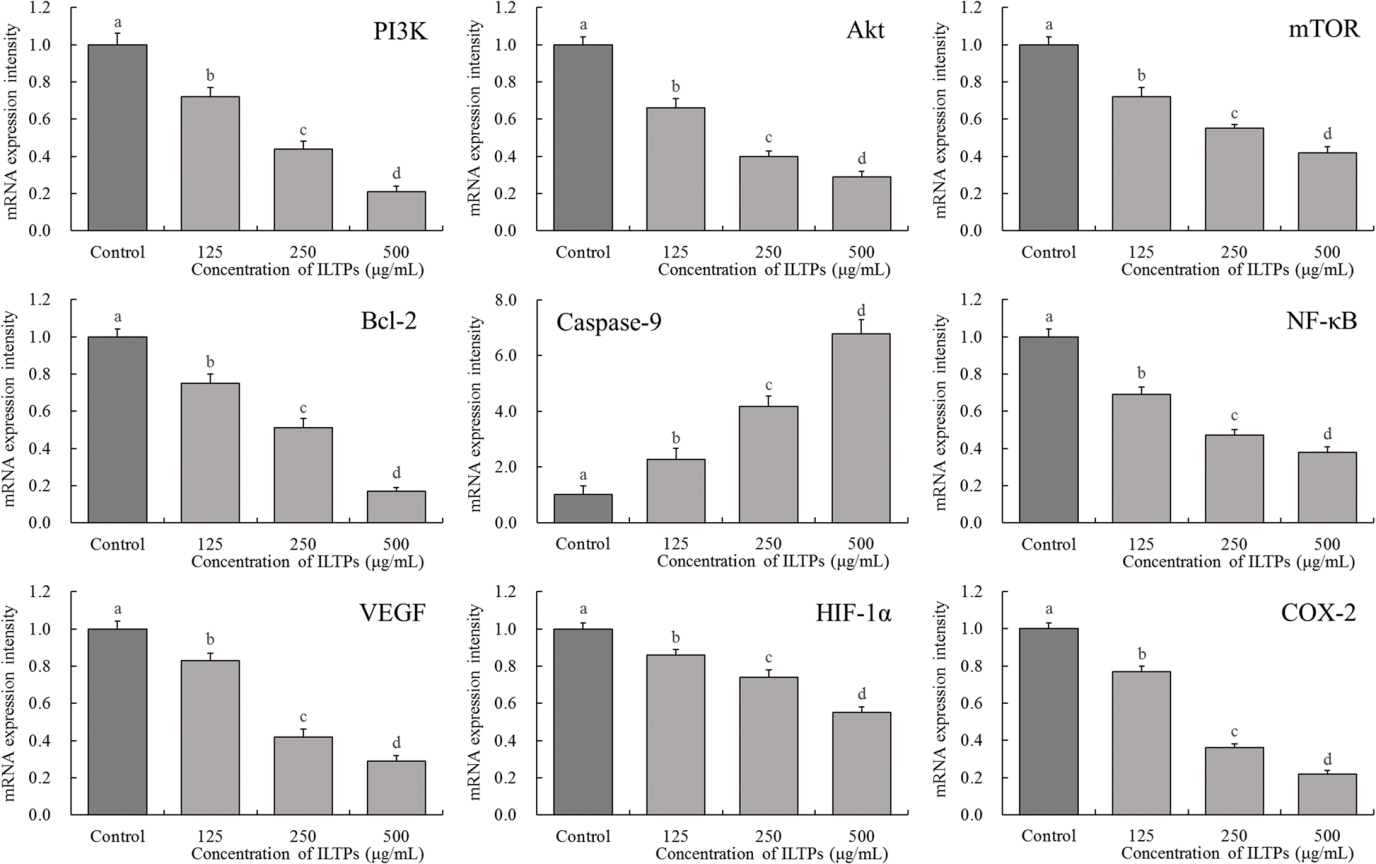
Effect of ILTPs on mRNA expression of A549 lung cancer cells (*n*=3). Duncan multiple range test showed that a-d of different letters showed significant difference in the mean value of each group (*P* < 0.05), the same letter indicates that there is no significant difference in the average value of each group (*P* > 0.05)

As shown in Fig. 5, A549 cells in the control group exhibited the weakest mRNA expression of caspase-9, while the strongest mRNA expression of PI3K, Akt, mTOR, B-cell lymphoma-2 (Bcl-2), nuclear factor-κB (NF-κB), vascular endothelial growth factor (VEGF), hypoxia-inducible factor-1 alpha (HIF-1α), and cyclooxygenase-2 (COX-2). Moreover, ILTPs was able to downregulate the expression of PI3K, Akt, mTOR, Bcl-2, NF-κB, VEGF, HIF-1α, and COX-2 and upregulate the expression caspase-9 in A549 cells. Compared with the control group, the expression of caspase-9 was stronger, while that of PI3K, Akt, mTOR, Bcl-2, NF-κB, VEGF, HIF-1α, and COX-2 decreased as the concentration of ILTPs increased.

### Expression of PI3K and Akt protein in A549 cells

**Fig. 6:**
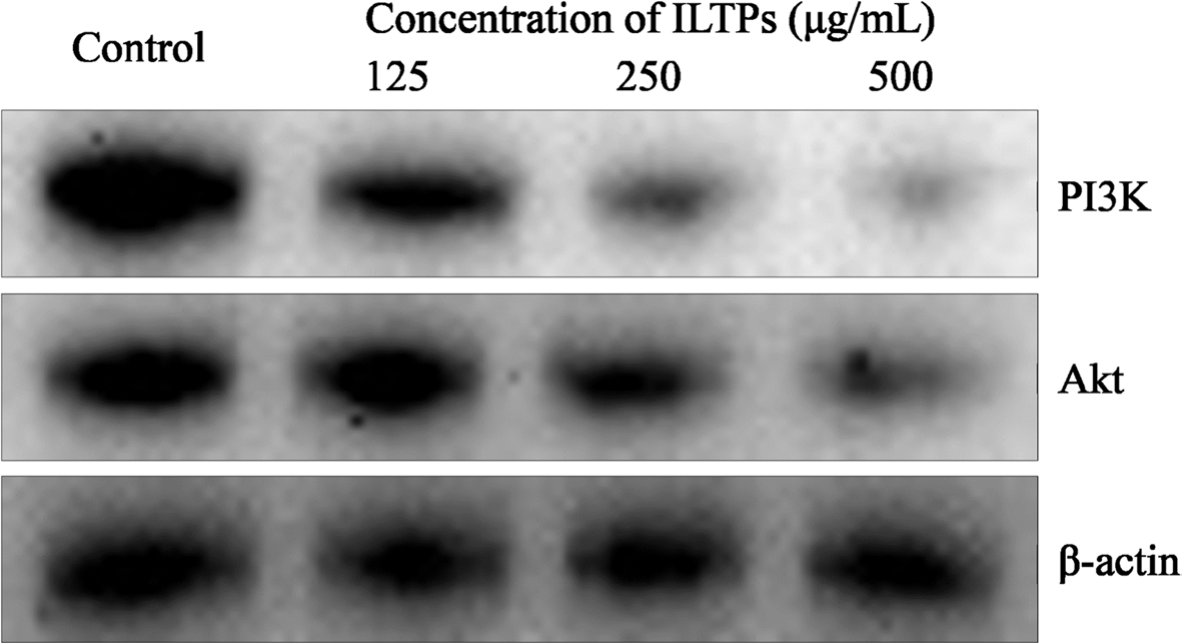
Effect of ILTPs on protein expression of A549 lung cancer cells (*n*=3)

As shown in Fig. 6, the A549 cells in the control group showed the strongest protein expression of PI3K and Akt . After treatment with ILTPs, the protein expression of PI3K and Akt decreased, and with the increase in the ILTP concentration, the protein expression of PI3K and Akt became weaker.

## Discussion

The main causes of cell death are apoptosis and cytotoxicity. ILTPs can induce apoptosis and death of cancer cells, but they have low toxicity and little effect on normal cell proliferation [18]. The present study demonstrated that ILTPs significantly inhibited only the proliferation of lung cancer cells, and did not affect normal lung tissue cells. Thus, ILTPs have the potential to be used as an active substance to inhibit lung cancer.

Under normal physiological conditions, only small amounts of LDH are present in body fluid, and intracellular LDH is released only after cell membranes are damaged. When the cancer cells are destroyed and die down, the LDH level in the culture medium significantly increases [19]. In this study, the LDH levels in the cell culture medium increased after lung cancer cells were treated with ILTPs, which showed that the ILTPs inhibited the proliferation of cancer cells and destroyed them. ROS can destroy normal tissues and DNA, leading to tissue lesions, which may be a contributing factor to cancer. In addition, a previous study showed that the ROS levels in cancer cells were higher than those of normal cells, and high levels of ROS might promote the growth and proliferation of cancer cells [20]. ILTPs have antioxidant and free-radical scavenging activity to protect the body [1]. The present study also showed similar results, where the ROS level of A549 cells was high, and after treatment with ILTPs, they might exert the ability to inhibit the high level of ROS in cancer cells. The ROS level of cancer cells decreased, subsequently inhibiting their negative effects.

The PI3K/Akt signaling pathway is an important cancer pathway because it can promote the growth and survival of cancer cells [21]. mTOR is a key kinase downstream of PI3K/Akt, and it plays a role in regulating the growth, proliferation, survival, and even metastasis of cancer cells [22]. The expression of PI3K, Akt, and mTOR inhibits pro-apoptotic factors and activates anti-apoptotic factors to promote the occurrence and development of cancer. PI3K/Akt-mTOR inhibits the activity of pro-apoptotic members and activates anti-apoptotic members through phosphorylation [23–25]. In addition, some studies showed that oxidative stress could activate PI3K/Akt [26, 27]. In this study, ILTPs effectively inhibited the expression of PI3K, Akt, and mTOR in the PI3K/Akt pathway. They also inhibited the PI3K/Akt pathway activated by oxidative stress, subsequently inhibiting the growth and proliferation of A549 cells.

In PI3K/Akt signaling activation, Akt activation is related to apoptosis, which, in turn, is related to the Bcl-2 family. The BAD of the Bcl-2 family can form dimers with Bcl-xL and promote cell apoptosis through signal transduction. Activated Akt can phosphorylate the ser136 site on BAD, thus preventing the apoptotic signal transmitted by BAD. After PI3K inhibitor transplantation, Akt-induced BAD phosphorylation is blocked [28]. Akt can also promote cell growth and inhibit cell apoptosis after the phosphorylation of NF-κB and activation of NF-κB in the nucleus [29]. When the cell is damaged, secondary damage to the mitochondrial membrane releases caspase and cytochrome *c*, and activates the cell death process through signal transmission. Activated Akt not only inhibits the release of apoptotic factors and cytochrome *c* but also inactivates caspase-9 by phosphorylating ser 196, and thus blocking its pro-apoptotic pathway [30]. After affecting the PI3K/Akt pathways, ILTPs also influence Bcl-2, NF-κB, and caspase-9 expression, thereby promoting cancer cell apoptosis.

PI3K joined the vascular endothelial signaling pathway of VEGF action via the activation of the PI3K/Akt pathway by forming a complex with E-cadherin, β-catenin, and VEGFR-12. Hypoxia and other factors, such as growth factors and insulin, can act on membrane surface receptors to induce the expression of HIF-1α, which prompts the translational expression of downstream angiogenic genes such as VEGF [31]. The activation of the PI3K/Akt pathway, which upregulates HIF-1α through multiple pathways, drives the expression of VEGF to enable the migration of endothelial cells to form a neovasculature network, so as to promote the growth and metastasis of cancer cells [32, 33]. VEGF, a major regulatory factor involved in angiogenesis, binds to endothelial cells in a targeted manner to promote vascular endothelial cell growth, increase their permeability, and then generate new blood vessels [33]. COX-2 is able to influence endothelial cell movement and the generation of new blood vessels, and the activation of the PI3K/Akt signaling pathway can upregulate COX-2, which in turn is involved in tumor angiogenesis [34]. On the contrary, ILTPs function to control cancer cell metastasis and proliferation by regulating the expression of VEGF, HIF-1α, and COX-2 under the influence of angiogenesis.

Rutin, kaempferol, and isochlorogenic acids A, B, and C are all polyphenolic antioxidant substances, and studies have shown that these compounds also exert some anticancer effects [35–39]. Rutin and kaempferol, as the main effective chemicals contained in some food products, also showed some interventional effects on lung cancer [40, 41]. The combined effect of these five compounds was responsible for the inhibitory effect by ILTPs on A549 cells *in vitro*, including the interventional effect on the pathway.

## Conclusions

This study found that ILTPs had little effect on normal lung tissue cells cultured *in vitro* but could inhibit cancer cell proliferation and cause A549 cells to undergo apoptotic death. ILTPs also played a role in regulating lung cancer cell apoptosis at the molecular level by regulating the expression of the PI3K/Akt signaling pathway. Five important polyphenol compounds contained in ILTPs contributed to these effects. The present study only preliminarily verified the effect and mechanism of ILTPs on lung cancer through *in vitro* experiments, and the effects and mechanisms that play a role in animals need to be explored in further studies.

## Supplementary Information

**Additional file 1.** Original strip of PI3K.

**Additional file 2.** Original strip of Akt.

**Additional file 3.** Original strip of β-actin.

## Data Availability

The western blot original strip in the supplementary material (Additional files 1, 2 and 3).

## Abbreviations

ILTPs: *Ilex latifolia* Thunb. polyphenols
PI3K: phosphatidylinositol 3-kinase
Akt: protein kinase B
MTT: 3-(4,5-dimethylazol-2-yl)-2,5-diphenyltetrazolium bromide
qPCR: quantitative polymerase chain reaction
HPLC: high-performance liquid chromatography
LDH: lactate dehydrogenase
ROS: reactive oxygen species
mTOR: mammalian target of rapamycin
Bcl-2: B-cell lymphoma-2
NF-κB: nuclear factor-κB
VEGF: vascular endothelial growth factor
HIF-1α: hypoxia-inducible factor-1 alpha
COX-2: cyclooxygenase-2.
