# Supplementary material for: Polyphenols in Ilex latifolia Thunb. inhibit human lung cancer cell line A549 by regulation of the PI3K-Akt signaling pathway
Source: BMC Complement Med Ther. 2022 Mar 23;22:85. doi: 10.1186/s12906-022-03568-3 (PMC8943935; doi:10.1186/s12906-022-03568-3)
Supplement: Supplementary file 2 — Additional file 2. Original strip of Akt. [file 12906_2022_3568_MOESM2_ESM.docx]

Raw gel image file of Akt.
